# Supplementary material for: Adherence monitoring methods to measure virological failure in people living with HIV on long-term antiretroviral therapy in Uganda
Source: PLOS Glob Public Health. 2022 Dec 30;2(12):e0000569. doi: 10.1371/journal.pgph.0000569 (PMC10021796; doi:10.1371/journal.pgph.0000569)
Supplement: S1 File — Table A: Association of ART adherence and virologic failure, adjusting for baseline patients’ characteristics (estimation of missing data on covariates by multiple imputation using chained equations. Table B: Association of ART adherence and virologic failure, adjusting for baseline patients’ characteristics (assuming all censored events i.e. deaths, LTFU, transferred out as virologic failures)–worst-case scenario. (DOCX) [file pgph.0000569.s001.docx]

**SENSITIVITY ANALYSES**

**Table A: Association of ART adherence and virologic failure, adjusting for baseline patients’ characteristics (estimation of missing data on covariates by multiple imputation using chained equations)**

| Factor | Model 1  ART adherence measured as 30-day self-report  (as main exposure) (n900) | | Model 2  ART adherence measured as Self-reported  (as main exposure) (n=900) | | Model 3  ART adherence measure Appointment keeping (as main exposure)  (n=900) | |
| --- | --- | --- | --- | --- | --- | --- |
| ART adherence (time-updated) | **aHR (95%CI)** | **P value** | **aHR (95%CI)** | **P value** | **aHR (95%CI)** | **P value** |
| Non-adherent | 1 |  | 1 |  | 1 |  |
| Adherent | 0.14 (0.05 – 0.35) | <0.001 | 0.35 (0.05 – 2.68) | 0.314 | 3.66 (0.35 – 37.77) | 0.447 |
| Other factors adjusted |  |  |  |  |  |  |
| Sex |  |  |  |  |  |  |
| Male | 1 |  | 1 |  | 1 |  |
| Female | 1.92 (0.68 – 5.36) | 0.215 | 1.93 (0.69 – 5.40) | 0.210 | 1.88 (0.67 – 5.27) | 0.231 |
| CD4 count at cohort registration (cells/mL) |  |  |  |  |  |  |
| <200 | 1 |  | 1 |  | 1 |  |
| ≥200 | 0.27 (0.08 – 0.92) | 0.036 | 0.23 (0.07 – 0.79) | 0.019 | 0.23 (0.07 – 0.78) | 0.018 |
| Employment status |  |  |  |  |  |  |
| Unemployed | 1 |  | 1 |  | 1 |  |
| Employed | 0.54 (0.21 – 1.36) | 0.190 | 0.50 (0.20 – 1.26) | 0.142 | 0.51 (0.20 – 1.30) | 0.158 |

**Footnote:** ART adherence modelled as time-dependent covariates in all models. Analysis performed on complete data (n=900) after multiple imputation of missing data on covariates. **aHR** denotes adjusted hazard ratio from Cox proportional hazard regression models, **CI** denotes confidence interval. Apart from adherence (the main exposure), in the adjusted models we Included only covariates with P value<0.2 at unadjusted model. Age groups, marital status and household monthly income had P values>0.2 at un adjusted Cox models, and thus were excluded from adjusted models.

**Table B: Association of ART adherence and virologic failure, adjusting for baseline patients’ characteristics (assuming all censored events i.e. deaths, LTFU, transferred out as virologic failures) – worst-case scenario**

| Factor | Model 1  ART adherence measured as 30-day self-report  (as main exposure) (n900) | | Model 2  ART adherence measured as Self-reported  (as main exposure) (n=900) | | Model 3  ART adherence measure Appointment keeping (as main exposure)  (n=900) | |
| --- | --- | --- | --- | --- | --- | --- |
| ART adherence (time-updated) | **aHR (95%CI)** | **P value** | **aHR (95%CI)** | **P value** | **aHR (95%CI)** | **P value** |
| Non-adherent | 1 |  | 1 |  | 1 |  |
| Adherent | 0.13 (0.06 – 0.31) | <0.001 | 0.46 (0.06 – 3.46) | 0.452 | 2.27 (0.27 – 18.83) | 0.447 |
| Other factors adjusted |  |  |  |  |  |  |
| Sex |  |  |  |  |  |  |
| Male | 1 |  | 1 |  | 1 |  |
| Female | 1.41 (0.57 – 3.46) | 0.456 | 1.57 (0.64 – 3.83) | 0.210 | 2.84 (0.80 – 10.06) | 0.106 |
| CD4 count at cohort registration (cells/mL) |  |  |  |  |  |  |
| <200 | 1 |  | 1 |  | 1 |  |
| ≥200 | 0.34 (0.10 – 1.15) | 0.083 | 0.34 (0.10 – 1.14) | 0.081 | 0.22 (0.06 – 0.76) | 0.017 |
| Employment status |  |  |  |  |  |  |
| Unemployed | 1 |  | 1 |  | 1 |  |
| Employed | 0.42 (0.18 – 09.8) | 0.045 | 0.43 (0.19 – 0.99) | 0.050 | 0.6021 (0.20 – 1.76) | 0.353 |

**Footnote:** ART adherence modelled as time-dependent covariates in all models. Analysis performed on complete data (n=900) after multiple imputation of missing data on covariates. **aHR** denotes adjusted hazard ratio from Cox proportional hazard regression models, **CI** denotes confidence interval. Apart from adherence (the main exposure), in the adjusted models we Included only covariates with P value<0.2 at unadjusted model. Age groups, marital status and household monthly income had P values>0.2 at un adjusted Cox models, and thus were excluded from adjusted models.

Assuming all censored participants (deaths, lost to follow-up and transferred-outs) achieved virologic failure: Incidence risk 10.4% (95%CI=8.6%, 12.6%) over 15 years, and incidence rate 19.45 (95%CI=15.97,23.92) per 1000 person-years.
